# Sensitive or sentient—a painful debate

**DOI:** 10.1007/s00709-021-01621-5

**Published:** 2021-02-13

**Authors:** Peter Nick

**Affiliations:** Botanical Institute, Karlsruhe Institute of Technology, Karlsruhe, Germany

In his short story “The Sound Machine”, Roald Dahl (1949) lets his hero, Klausner, develop a machine enabling to perceive the sounds of plants. When he puts on his headphones, he can hear the painful shrieks, while his neighbour prunes his roses. To test his machine further, Klausner cuts a wound into a tree and gets shocked about the moaning sound coming from the wounded tree. Terrified, he calls his doctor to witness, what he has heard in his headphones and, thus, to relieve him from the suspicion that he fell prone to insanity.

This story might appear as an ironic prelude for a controversial debate that has been shaking the plant community since a couple of years. Can plants develop emotions, intelligence and sentience just in the way as we humans do, and *Homo sapiens*, intoxicated by anthropocentric arrogance, is just too tunnel-viewed to perceive this? Alternatively, are we unable to realise these features, because they are simply absent in plants? Both viewpoints exist and they seem to mark a canyon that is difficult to bridge. However, it might be useful to let both positions collide—development is usually requiring polarities to advance, and so does science.

A central topic in this controversy is the question, whether plants are able to feel pain. To answer this question is far from trivial. We know quite well, whether *we ourselves* feel pain. However, already the question, whether our fellow humans *feel* pain and to what degree their pain corresponds to ours, is difficult to judge. We help ourselves by assessing the *expression* of pain, making use of mirror neurons that allow us and other primates to mimic actions of others and, thus, to recapitulate their emotions from the simulation of their facial expression (Singer et al. 2004). Empathy comes from resonance, and resonance depends on similarity (this is true for physics as well as it is true for psychology). As soon as the similarity decreases, our ability to feel empathy rapidly drops as well. The pain of a cute dog will still arise our spontaneous sympathy, while the squirming worm on our fishing hook almost escapes our attention. To perceive, whether and how plants respond to damage, requires already sophisticated experimentation, not so different from Klausner’s sound machine.

As special and even esoteric this debate may seem, it is nothing else than a very common theme in biology: whether two, obviously different, phenomena are just different manifestations of the same thing, or whether they are so different, because there is nothing underneath that could be called “the same thing”, is a salient question in evolutionary theory. Things can be alike without having a common essence (convergence), or they can be alike, because they derive from the same origin (homology). To deduce homology, requires *comparing* the two items. As a first step of comparison, we have *to align* them. This holds true for the sequence of two proteins, whose homology we want to infer, or for the two skeletons, from which we can deduce (when we lay them out side by side) that the arm of a human corresponds to the wing of a bird, although both structures look quite different (Belon du Mans 1555). Two contributions to the current issue, a critical review by Robinson et al. (2021), and a response by Baluška and Yokawa (2021), are doing nothing else than leading a debate about convergence versus homology on the topic of pain in plants.

A central argument in the debate runs as follows: anaesthetics can silence consciousness and perception in humans, including pain. They also can silence the responses of plants to exogenous factors, including responses to damage. Therefore, these silenced responses to damage correspond to the silenced responses of humans to damage and, thus, correspond to pain.

In their review, Robinson et al. (2021) dissect this argument. They first concede that anaesthetics do evoke effects in plants, including both local responses, such as changes of ion-channel activity, but also systemic responses, such as jasmonate-based signalling. However, they emphasise that most anaesthetics affect multiple targets (that are also present in other life forms) and that the specific effect on humans originates from their modulation of neural activity. If this specificity derives from the presence of neurons, the absence of neurons would argue against a homology, but in favour of a convergence. To substantiate this point, they systematically define the phenomenon of pain and dissect it into different components, as there are a discriminative, a cognitive, an affective, a stress- and fear-related and a physiological element. They also elaborate how these components associate with different regions of the brain, rendering pain a very complex, in the true sense of the word, holistic, experience. They further point out that pain can occur even without damage. Thus, the ability to sense damage (nociception) is not the same thing as the experience of pain.

After having defined the concept of pain and the components being part of it, they, basically, develop a classical evolutionary argument, asking, to what extent these components are present or absent, when one moves away from humans. For the mammals, this is straightforward, because they possess all components present also in the human pain system. These include nociceptors, pain pathways, subcortical brain regions and the various pain-related areas of the cerebral cortex. This turns out to be already less straightforward in the non-mammalian vertebrates that lack a cortex. The notion that these vertebrates should therefore not be able to experience pain would contradict common sense (and by the way, all legislation of civilised countries). The argument is here that the subcortical pain regions are still present and that other structures functionally replace the cortical components of the mammalian pain system, such as the cerebral hemispheres of birds.

The authors step now further towards the invertebrates. Here, the neural system has developed independently, and homology holds valid only for the nociceptors. Here, the authors use a convergence argument stating that one needs to consider pain-indicating behaviour for assessment. The criteria are learning from painful experience to avoid damage, or places, where they had experienced damage earlier, curative behaviour such as guarding wounds, or the self-delivery of analgesic pain relievers. They conclude that, among the invertebrates, arthropods and cephalopods meet these criteria, and therefore, are life forms definitely capable of pain experience (which is in line with most legislation on animal experimentation). The authors argue now that operant learning or conditioned place aversion are absent in plants. Nevertheless, they go on defining two criteria required for pain experience. The first is the presence of nociceptors. While there are no obvious plant candidates for nociceptive cells, there exist ion channels as those participating in animal nociception, and there exist mechanosensitive systems in both life forms. To what extent these are fulfilling the same function remains an open question. To what extent they are homologues remains an even more open question. However, the authors do not bluntly exclude this possibility. They rather focus on the second argument that plants lack a system to integrate and experience damage, because they lack neurons and a brain. While they concede that, in plants, damage can evoke long-distance chemical and electrical signals as well, they dismiss them as analogues to the pain system of animals. They spell this out in more detail, for instance, by questioning, whether plant homologues of the neural GABA receptors are playing the same functions (and thus, might serve as homology marks for a plant neural system), or whether plant compounds produced in response to wounding, such as divinyl ether or ethylene are self-anaesthetics. In summary, they arrive at the conclusion that the molecular, cellular and supercellular details of damage responses are different in both essence and functional context, and hence not homologous and not even convergent. Using the terminology of Remane (1952), who defined criteria to infer homology, the damage response of plants would not be homologous to the pain system of animals, because it does not meet the criterion of specific quality.

What is now the viewpoint of Baluška and Yokawa (2021) on plant anaesthetics? They begin with a short historic overview on the position of plants in the system of nature—while first settled somewhere at the margin of life, close to the minerals (Aristotle, Kant), they were later found to be endowed with complex sensory and adaptive systems. Around fifteen years ago, the authors have coined the concept of a plant neurobiology, drawing an analogy between membrane potentials and voltage-gated ion channels that are also present in plants (as well as in all other life forms) and the electric signalling of neurons. The existence of action potentials in some (specialised) plants, the endocytic recycling of vesicles as it is also found in neural synapses, and intercellular adhesion domains that compare to synaptic connections serve as criteria to underpin the concept that plants, while not possessing a neural system, possess something that acts like a neural system. They admit that this comparison raises terminological issues, because terms that describe brain-related or at least neuron-related phenomena move out from their original context. To elaborate their analogy further, they emphasise that all living organisms need sensory systems and the ability to adapt to their environment. In addition, they list agency, cognition and behaviour as fundamental attributes of life (contesting that the manifestation of these properties differs between different life forms). After laying out this conceptual framework, they are then investigating the effect of anaesthetics upon plants.

One of the molecular targets for anaesthetics are neural lipid rafts, harbouring specific phospholipase D members, important signalling hubs that are also present in plants and associate with plant lipid rafts. They propose that fluorescent markers and mutant lines might help to see a potential effect of anaesthetics, which would represent a testable implication of their hypothesis. They continue in pointing out that plant-derived compounds act as anaesthetics (which would correspond to the analgesic pain-reliever criterion to assess pain expression in invertebrates). As one example, they use the stress hormone ethylene, which was in use to anesthesise humans up to the 1930. This interpretation gets strong criticism by Robinson et al. (2021) that emphasise that ethylene is acting as a hormone, rather than as an anaesthetic, and that the fact that ethylene has anaesthetic effects in humans does not mean that it conveys this function in plants. In fact, while plants do produce numerous compounds with analgesic effects (which is an important aspect of the medical use of plants for human medicine), many of those compounds target to attacking herbivores (prominent examples are the poppy opiates or the hemp cannabinoids, where the plant compound mimics endogenous analgetic compounds of the animal), rather than upon the plant itself. For their argument, the case of methyl salicylate is interesting, though, because this compound acts antagonistic to jasmonate signalling and, thus, can suppress the wounding response.

They address then the case of plant GABA receptors, defending their interpretation that these might serve as homology marks for a “neural” nature of plant cells. They point out that plants and animals share the antagonism between glutamate and GABA on membrane voltage and that glutamate (one of the targets for anaesthetics) takes part in modulating action potentials in plants. Further, they mention that the alkaloid berberine can interfere with glutamate-dependent signalling in neurons (they do not discuss whether this is a further mechanism to deter herbivores, or whether they think that berberine acts as a kind of plant endorphine). This line of argument would again be of the specific quality type (Remane 1952). However, in contrast to Robinson et al. (2021), they conclude that the details of GABA and glutamate signalling would support a homology of plant and neural cells, proposing, “neuron-like electrical long distance […] assembles plant bodies into coherent units acting as single cognitive selves”. They conclude by inverting their historic starting point and state that the notion of plants as life forms void of sensory and cognitive faculties would have serious consequences for our way to deal with trees and forests.

While both contributions use homology arguments (specific quality) to compare human pain experience with plant nociception, they arrive at different conclusions. Editorial neutrality prevents to take a stand in this debate, but it may be helpful to formulate two questions, as well as to propose a terminological specification:

Pain is a holistic experience as pointed out in detail by Robinson et al. (2021). This requires that there is some “body”, i.e., an experiencing entity. In humans and in animals, the concept of a body means—it is distinct, defined and endowed with a strongly hierarchical structure, whose genesis depends on a tight genetic control and is mostly independent from environmental variability. Has the “body” of a plant really the same essence, or does a plant “body” resemble a collective system composed of relatively autonomous cells that can flexibly respond to the environment through dynamic self-organisation?

Pain is also a temporal experience—the time scale of animals that move and use movements as signals (we use the term behaviour to describe this phenomenon) is different from plant time that gets its structure from developmental responses. The cases, where these responses are fast enough to become perceived by us, are specialised—the venus fly trap or the mimosa leaf have attracted so much attention, because they deviate from that what plants normally do. While electrical phenomena occur in all life forms, because they are the natural consequence of selective membrane permeability, this does not necessarily imply that these electrical phenomena act as signals. Whether an event or a molecule becomes a signal, depends not on its own essence, but on the functional context, as Karl Bühler (1934) pointed out in his Organon model. It would be an important question to discuss, whether electrical phenomena have the same functional context in animals and in plants.

A terminological definition might also help to render the debate more fruitful. To create an opposition between plants as “senseless automata” and plants as “cognitive selves” does not leave much space for any bridging positions in-between. There is probably little dispute about the fact that plants are very successful life forms, because they have evolved complex, flexible and highly efficient signalling. Thus, they are definitely *sensitive* beings. It would be helpful to separate this from the discussion, whether they are *sentient* beings.

What would Klausner contribute to this debate? We will never know—in a typical Dahlian move, he tries to make the doctor hear what he has heard and takes a second swing, when a large branch crashes down and destroys the machine. Thus, the doctor has to remain agnostic. Nevertheless, he accepts Klausner’s wish to care the wounds with iodine.
